# Engineered SERPINE1 Overexpression Activates the pp53/p21 Pathway and Promotes a Growth-Restricted, Migratory Phenotype in KRAS-Transformed Renal Proximal Tubular Cells

**DOI:** 10.3390/biom16101517

**Published:** 2026-10-19

**Authors:** Naziba Nuha, Craig E. Higgins, Stephen P. Higgins, Rohan Samarakoon, Paul J. Higgins

**Affiliations:** Department of Regenerative & Cancer Cell Biology, Albany Medical College, Albany, NY 12208, USA; nuhan@amc.edu (N.N.); craig.evan.higgins@gmail.com (C.E.H.); higgins@amc.edu (S.P.H.); samarar@amc.edu (R.S.)

**Keywords:** SERPINE1, PAI-1, KRAS, renal epithelial cells, migration, p53, p21

## Abstract

Oncogenic Ras signaling drives cell growth and tumor progression through acquired changes in gene expression that promote the malignant phenotype. One such gene, SERPINE1, which encodes plasminogen activator inhibitor-1 (PAI-1), is a poor prognostic biomarker in human cancers and a p53 target gene. This study utilizes v-KRAS-transformed renal epithelial (KNRK) cells to define how PAI-1 overexpression influences cell morphology, growth dynamics, and migratory behavior. KNRK cells were engineered to express PAI-1 using a CMV-driven lentiviral expression vector, with a CMV drug resistance only vector as a control, followed by stable selection with puromycin. Cellular morphology and growth characteristics were assessed by microscopic imaging and cell density analysis; cell migration was evaluated using a scratch-wound assay. Western blotting and immunofluorescence served to assess expression and subcellular localization of PAI-1, p21, and p53 in CMV-control and PAI-1 cells. CMV-PAI-1 cells exhibited a shift to a mesenchymal, more pleomorphic and motile phenotype, with increased spreading, irregular borders and decreased cell–cell adhesion. Wound closure in CMV-PAI-1 cells was significantly increased compared to CMV-control cells (*p* < 0.001), indicating enhanced migratory capacity, with prominent deposition of PAI-1 into cellular migration trails. CMV-PAI-1 cells had a reduced growth rate, an approximately 4-fold increase in p-p53 and a shift in p21 from a perinuclear localization in controls to predominantly cytoplasmic in CMV-PAI-1 cells. These findings highlight PAI-1 as a potential driver of mesenchymal cell traits in KRAS-transformed renal epithelial cells.

## 1. Introduction

Mutations in the KRAS oncogene are among the most prevalent alterations in human cancers and are strongly associated with poor prognosis, therapeutic resistance, and metastatic spread [1,2,3]. KRAS mutations at the G12 residue (G12D, G12V, G12C, and G12R) are evident in most pancreatic tumors (>85%) closely followed by colorectal cancers and cholangiocarcinomas [4,5,6]. Mutant KRAS constitutively activates downstream PI3K/AKT and MAPK pathways driving cellular proliferation, increased motility and tumor cell survival while promoting development of a dense, highly fibrotic (desmoplastic) stromal microenvironment [6,7].

Emerging transcriptomic and proteomic evidence implicates the clade E, member 1 serine protease inhibitor SERPINE1 (also known as plasminogen activator inhibitor-1 or PAI-1) as a major hub gene and critical effector of tumor progression and the desmoplastic reaction [8,9,10,11,12,13,14]. PAI-1 is traditionally known for its role in inhibiting fibrinolysis by blocking tissue-type and urokinase plasminogen activators [15]. Recent studies, however, highlight its involvement in modulating the tumor microenvironment (TME), where it regulates extracellular matrix dynamics by controlling the spatial/temporal process of stromal remodeling, and altering intracellular signaling pathways [16,17] PAI-1 expression is tightly regulated by cytokines and stress signals, notably p53 and TGF-β1, underscoring its potential integration into key oncogenic networks [10,11,14,18,19]. Mutant KRAS pancreatic and colorectal tumors, moreover, commonly harbor TP53 mutations. Loss of wild-type p53 results in a significant decline in miR-34a, a microRNA that normally suppresses SERPINE1 expression [20].

In normal cells, PAI-1 is part of a protective cellular response to damage, inducing cell cycle arrest and senescence in the presence of genotoxic stress through the p53/p21 pathway [21,22]. However, the role of PAI-1 is paradoxical in the context of cancer, where elevated expression correlates with tumor aggressiveness, metastasis, and poor clinical outcomes. Indeed, PAI-1 promotes cellular migration and tissue invasion by interacting with various cell surface receptors (e.g., the low-density lipoprotein receptor-related protein 1 [LRP1], integrins, urokinase plasminogen activator and its receptor [uPA/uPAR], and the epidermal growth factor receptor [EGFR]) as well as by regulating stromal remodeling [8,10,11,23,24,25]. This PAI-1 interactome comprises a complex network of extracellular matrix (ECM) proteins, cellular receptors, and intracellular signaling molecules and raises the likely possibility that increased PAI-1 expression may positively impact the malignant phenotype, either directly or through intermediary signaling cascades. Supportive of this is the recent finding that human HK-2 renal epithelial cells engineered to synthesize high levels of PAI-1 underwent dedifferentiation (E-cadherin loss, gain of vimentin expression), G2/M arrest (elevated levels of p-Histone3 and p21), p53 upregulation and increased expression of desmoplastic ECM molecules [26]. This study aims to define the morphologic and motile responses of renal epithelial cells harboring an oncogenic KRAS mutant to characterize the phenotypic and molecular consequences of PAI-1 dysregulation.

## 2. Materials and Methods

### 2.1. Cell Lines and Lentiviral Transduction

KNRK cells, a rat proximal tubular epithelial cell line stably transformed with the Kirsten murine sarcoma virus (Ki-MSV) bearing a constitutively active v-KRAS gene, were cultured in Dulbecco’s Modified Eagle Medium (DMEM) supplemented with 10% fetal bovine serum (FBS) and 1% penicillin–streptomycin (ThermoFisher, Waltham, MA, USA), and maintained at 37 °C in a humidified 5% CO_2_ incubator. KNRK cells were transduced with cytomegalovirus (CMV) promoter lentiviral vectors driving a puromycin or G418-resistance gene (control) or a CMV-PAI-1 expression construct. Lentiviral production used HEK293T cells with second-generation packaging vectors; viral supernatants were collected and filtered before use. KNRK cells were transduced in the presence of 8 μg/mL polybrene and selected with 2 μg/mL puromycin (or G418) for stable integration. Successful transduction and PAI-1 expression were confirmed by immunoblotting. HK-2 human proximal tubule epithelial cells and newborn rat keratinocytes (RK cells), engineered to express high PAI-1 levels, and PAI-1 knockdown 4HH cells were described previously [26,27,28,29]. RK cells were transfected to express a PAI-1-GFP construct as a fusion protein under inducible control of +800 bp of the wound-activated PAI-1 promoter [29].

### 2.2. Western Blot Analysis

Total cellular protein was extracted using RIPA buffer supplemented with protease and phosphatase inhibitors (Thermo Fisher Scientific). Protein concentrations were determined using a bicinchoninic acid (BCA) assay. Equal amounts of protein (30–50 μg) were separated on 10% SDS-PAGE gels and transferred to polyvinylidene difluoride (PVDF) membranes. Membranes were blocked with 5% bovine serum albumin (BSA) in Tris-buffered saline with 0.1% Tween-20 (TBST) and probed overnight at 4 °C with primary antibodies specific for PAI-1, phospho-p53 (Ser15), total p53 (Cell Signaling Technology, Danvers, MA, USA). The following day, membranes were washed and incubated with IRDye-conjugated secondary antibodies (LI-COR Biosciences, Lincoln, NE, USA), and fluorescence-based detection was performed using the Odyssey CLx Imaging System. Band intensities were quantified with Image Studio software 6.2 and normalized to the corresponding loading control.

### 2.3. Immunofluorescence Microscopy

For imaging studies, cells were seeded on sterile glass coverslips in 24-well plates, cultured to 60–70% confluence then fixed with 4% paraformaldehyde for 15 min at room temperature, followed by permeabilization with 0.1% Triton X-100 in phosphate-buffered saline (PBS) for 10 min. After blocking with 5% goat serum for 1 h, coverslips were incubated overnight at 4 °C with primary antibodies and washed, and Alexa Fluor-conjugated secondary antibodies (Thermo Fisher) were applied for 1 h at room temperature. Nuclei were counterstained with 4′,6-diamidino-2-phenylindole (DAPI) and coverslips mounted using ProLong Gold Antifade Reagent. Images were acquired using a Zeiss LSM 880 confocal microscope (Carl Zeiss, White Plains, NY, USA) with identical laser settings for all conditions. Image analysis and quantification utilized FIJI/ImageJ (v1.54u).

### 2.4. Statistical Analysis

Statistical comparisons for triplicate independent experiments among all experimental groups used one-way analysis of variance (ANOVA), followed by Tukey’s post hoc test for multiple comparisons. *p*-values less than 0.05 were considered statistically significant. GraphPad Prism 9 software was used for statistical analysis and plotting.

## 3. Results

**Figure 1:**
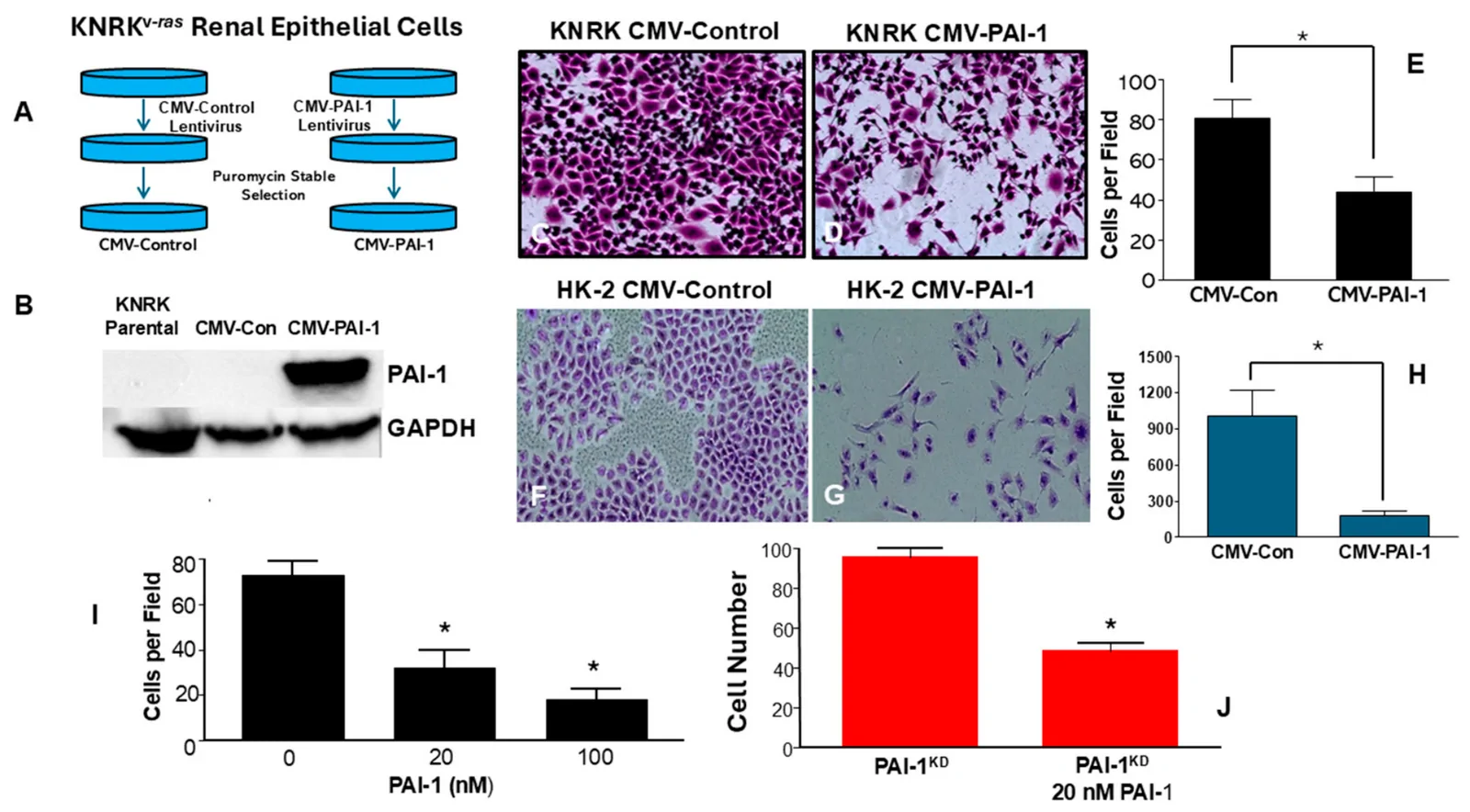
**Generation and characterization of stable PAI-1 expressing KNRK^v-RAS^ cells.** (**A**) KNRK cells were infected with lentiviral particles bearing a CMV-control vector or a CMV vector containing a PAI-1 cDNA insert (CMV-PAI-1) followed by puromycin selection to establish stable cell lines. (**B**) Western blotting confirmed the robust PAI-1 expression in CMV-PAI-1 transductants relative to KNRK parental or KNRK CMV-control cells. GAPDH served as a loading control. Brightfield images of CMV-control and CMV-PAI-1 KNRK (**C**,**D**) and HK-2 (**F**,**G**) cells illustrate the reduced cell density and scattered, mesenchymal phenotype of PAI-1 overexpressing cells. Microscopic quantitation confirmed the reduction in cell growth 5 days after seeding equivalent numbers of cells per culture for both KNRK (**E**) and HK-2 (**H**) CMV-PAI-1 transductants relative to CMV-controls. Exposure to the PAI-1 14-1B stable mutant protein at 20 and 100 nM significantly suppressed KNRK cell growth after a 5-day incubation (**I**). Recombinant PAI-1 14-1B also effectively inhibited proliferation of the PAI-1 knockdown 4HH cell line (**J**). * = *p* < 0.05; plots (**E**,**H**–**J**) are results of 3 independent experiments. Original Western blot images are provided in the Supplementary Materials.

Infection of KNRK^v-KRAS^ renal epithelial cells with lentiviruses encoding a CMV-driven puromycin-resistance gene (control) or a PAI-1 insert, followed by stable selection in puromycin, resulted in the generation of CMV-control and CMV-PAI-1 cell lines (Figure 1A). Western blotting confirmed the robust expression of PAI-1 by CMV-PAI-1 transductants compared to CMV-controls and parental KNRK cells (Figure 1B). An earlier study of KNRK cells engineered to express vector-driven PAI-1 indicated that the frequency of cells with cytoplasmic-to-nuclear ratios ≥ 2 among four clonal isolates varied from 16.0 ± 6.2 to 46.2 ± 8.6 suggesting a significant pleomorphic response to PAI-1 overexpression [28]. Consistent with this finding, considerable PAI-1-associated morphological variability was evident in KNRK and HK-2 CMV-PAI-1 populations relative to the corresponding CMV-control cells (compare Figure 1C,F with Figure 1D,G). The level of PAI-1 expression in CMV-PAI-1 KNRK cells was similar to that induced by TGF-β1 in mutant KRAS-transformed pancreatic and colorectal cell lines and HaCaT keratinocytes bearing a mutant HRAS gene (manuscript in preparation). The pleomorphic response typical of CMV-PAI-1 transduced KNRK and HK-2 renal epithelial cells was confirmed as due to the engineered expression of PAI-1 [26,28]. The final population density in both CMV-PAI-1 KNRK and HK-2 cells, moreover, was significantly reduced compared to CMV-controls; this response, however, as was the elaboration of a mesenchymal-type, spindle-shaped phenotype, was more prominent in the HK-2 background (Figure 1E,H). To further implicate PAI-1 in proliferative restriction, KNRK cells were cultured in medium containing 20 and 100 nM of the long half-life (t = 145 h) PAI-1 stable mutant 14-1B. Exposure to PAI-1 14-1B effectively suppressed KNRK cell growth in a concentration-dependent manner after a 5-day incubation period (Figure 1I). Recombinant PAI-1 14-1B, moreover, inhibited proliferation of the PAI-1 knockdown 4HH cell line (Figure 1J). Collectively, these findings are consistent with the known cytostatic effects of high level PAI-1 expression in particular cell types [21].

**Figure 2:**
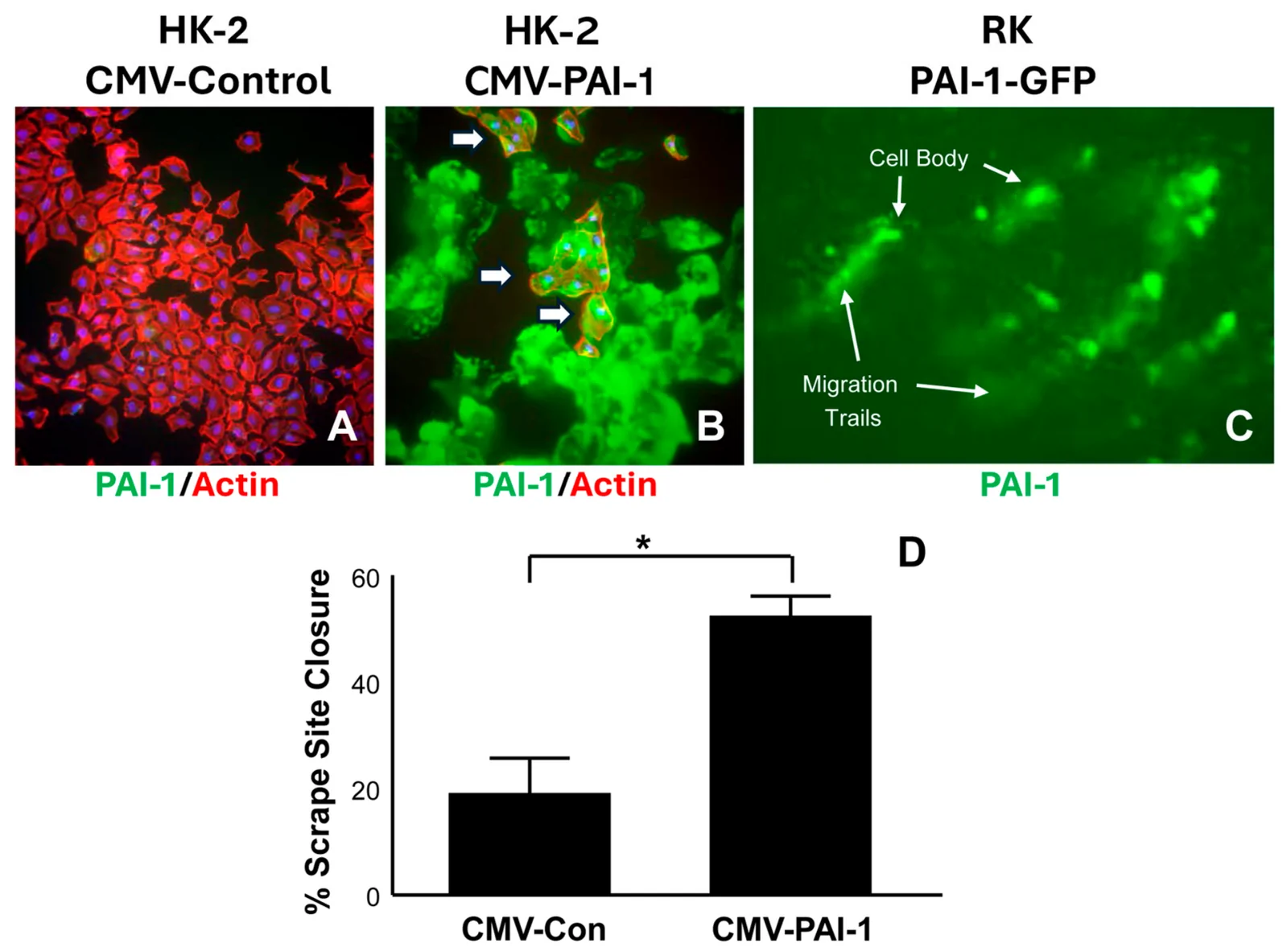
**PAI-1 expression confers a migratory phenotype in HK-2 and KNRK renal epithelial cells.** Unlike HK-2 CMV-controls, which are PAI-1^Low^ and relatively sessile (**A**), HK-1 CMV-PAI-1 cells are highly motile with abundant PAI-1 deposition into cellular migratory tracks (**B**). Thick arrows in (**B**) indicate individual cells. For comparison, Panel (**C**) illustrates the prominent PAI-1-decorated migration trails seen in rat keratinocytes (RK cells) engineered to express a PAI-1-GFP chimeric expression construct in which the full-length human PAI-1 coding sequence-GFP insert is under inducible control of +800 bp of the wound-activated PAI-1 promoter [29]. Quantitative assessments of the extent of scrape wound site closure 48 h after injury confirmed that KNRK CMV-PAI-1 cells were significantly more migratory than CMV-control populations. * = *p* < 0.05; plot (**D**) is the compilation of 3 independent experiments.

Prior analysis further confirmed a significant loss of the epithelial marker E-cadherin (>98% decrease) and robust upregulation of the mesenchymal proteins vimentin (>28-fold) and α-smooth muscle actin (α-SMA) (>2.8-fold), which correlated with increased cytoarchitectural reorganization in CMV-PAI-1 cells relative to control vector transductants [26]. Brightfield imaging revealed acquisition of a mesenchymal-like scattered morphology in the CMV-PAI-1 HK-2 and CMV-PAI-1 KNRK populations relative to the corresponding CMV-control cultures (Figure 1C,F vs. D,G) suggesting adaptation of a more motile phenotype. Immunofluorescence localization of PAI-1 in HK-2 cells confirmed that, unlike the rather sessile CMV-controls, CMV-PAI-1 cultures comprised rather sparce but highly motile cells with PAI-1 prominently deposited in cellular migratory tracks (Figure 2A,B), reminiscent of a similar finding in keratinocytes (Figure 1C and [29]). Similarly, the scrape wound assay established the significantly increased migratory response of PAI-1 overexpressing KNRK cells following monolayer injury (Figure 2D).

**Figure 3:**
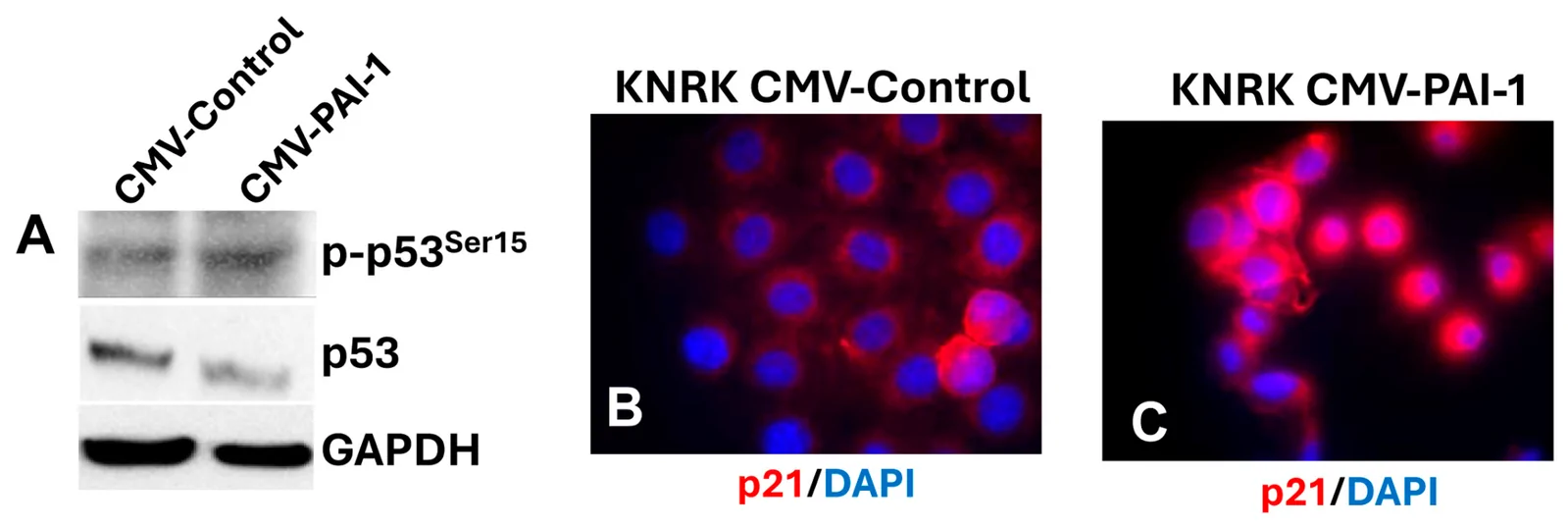
**Expression of phospho-p53 (p-p53) and p21 in CMV-control and CMV-PAI-1 cells.** (**A**) Western blotting indicated an approximately 4-fold increase in p-p53 in CMV-PAI-1 cells compared to CMV-controls when taking into account the lower level of total p53 in CMV-PAI-1 cells using GAPDH as a loading control. p21 immunolocalization in CMV-control cells was exclusively perinuclear (**B**) whereas in the PAI-1 overexpressing CMV-PAI-1 line p21 was more cytoplasmically distributed (**C**). Original Western blot images are provided in the Supplementary Materials.

Previous studies indicated that vector-driven PAI-1 expression results in p53, p-p53^Ser15^ and p21 upregulation in HK-2 renal epithelial cells that express a wild-type KRAS gene [26]. Since PAI-1 effectively inhibits proliferation of both wild-type and mutant KRAS cells (Figure 1E,H), it was important to assess the effect of PAI-1 overexpression on the p53/p21 axis in KNRK cells. Western blot quantitative analysis using Image Studio to compare the p53 and p-p53^Ser15^ band intensities in CMV-control vs. CMV-PAI-1 cells indicated that KNRK CMV-PAI-1 cells expressed approximately 4.0 ± 0.9-fold (*p* < 0.05) more p-p53^Ser15^ than the corresponding CMV-controls (Figure 3A). It was previously confirmed that HK-2 CMV-PAI-1 cells also produced significantly more (>10-fold; *p* < 0.05) p-p53^Ser15^ relative to the CMV-controls [26]. The increased levels p-p53^Ser15^ in CMV-PAI-1 populations correlated with a prominent cytoplasmic localization of the growth arrest gene p21, a major transcriptional target of p53 (Figure 3B,C).

## 4. Discussion

Several elements that comprise the plasmin-based pericellular proteolytic cascade, including uPA [30,31], uPAR [32], the LDL-receptor-related protein 1 (LRP1) [33] and PAI-1, are poor prognostic factors for patients with cancer, e.g., [34]. uPAR localizes to the surface of neoplastic cells and tumor-infiltrating macrophages and is prominent at the invasive tumor front, presumably promoting repetitive cell attachment/detachment mechanisms in support of cell migration and tissue invasion [12,35]. Upon uPA binding, uPAR activates several signaling pathways (e.g., MAPK, JAK/STAT, and PI3K/AKT/mTOR) through crosstalk with cell surface binding partners (e.g., PDGFR, LRP1, GPCRs [e.g., FPRL1], and αv-integrins) facilitating cancer cell growth, survival, migration, and metastasis [35,36].

The *SERPINE1* gene encodes the serine protease inhibitor clade E member 1 protein SERPINE1 also known as plasminogen activator inhibitor-1 (PAI-1). PAI-1 targets the tissue-type plasminogen activator (tPA) and urokinase PA (uPA) pericellular proteolytic systems thereby regulating extracellular matrix (ECM) and stromal remodeling, e.g., [17]. The role of PAI-1 in cancer biology, however, is more complex and not restricted to its anti-proteolytic properties [11,37,38]. Indeed, PAI-1 has emerged as a pan-cancer poor prognosis biomarker, a regulator of cell migration, tissue invasion and angiogenesis and a major contributor to tumor cell survival and drug resistance [8,33,38,39,40]. PAI-1 is expressed by malignant cells, endothelial elements and infiltrating cellular components in the tumor stroma including cancer-associated fibroblasts and tumor-associated macrophages [10,11,12,41,42,43,44]. PAI-1 is particularly evident in myofibroblastic-like cells at the invasive front, and increased expression levels of PAI-1 in the tumor microenvironment is a worse prognostic indicator in many cancers [10,11,12].

The morphological changes induced by engineered PAI-1 expression in KNRK and HK-2 renal cells mirror characteristics of an epithelial-to-mesenchymal transition [45,46] and, at least for CMV-PAI-1 HK-2 cells which have a wild-type KRAS gene, is consistent with earlier findings [26]. The significantly increased motility in CMV-PAI-1 KNRK and HK-2 cells, moreover, reflected a marked reduction in final population density. It is now apparent that PAI-1 has context-dependent effects on cell proliferation and can stimulate or suppress cell growth [13,14,47]. Indeed, PAI-1 is also not merely a marker of the senescent phenotype but is both necessary and sufficient for the induction of replicative senescence downstream of p53 [21,48,49,50]. PAI-1 is a major p53 target gene and PAI-1 overexpression is associated with a marked induction of p53 in both KNRK and HK-2 cells and in the redistribution of the p53 growth arrest target gene p21 [this paper and 26]. This last finding has pathophysiologic implications as cytoplasmic p21 appears to have oncogenic properties and promotes tumor cell survival, migration, metastasis and drug resistance [51,52,53,54,55]. It is also apparent that the cytoplasmic localization of p21 in KNRK CMV-PAI-1 cells is very similar to that seen in drug-resistant, poor prognosis, malignancies including testicular, colorectal and triple-negative breast cancers [54,56,57,58]. This dichotomy in PAI-1 function is not well understood and is further highlighted by its proliferative and pro-epithelial to mesenchymal effects on certain neoplastic cells while also stimulating entrance into a senescence phenotype.

## 5. Conclusions

PAI-1 coordinates signals between tumor cells, stromal cells, and immune cells to build a supportive environment for cancer growth and metastasis. PAI-1 is a pan-cancer poor prognosis biomarker, a regulator of cell migration, tissue invasion and angiogenesis and a major contributor to tumor cell survival and drug resistance. Elevated expression of PAI-1 correlates with tumor aggressiveness, metastasis, and poor clinical outcomes. This study highlights the morphologic and motile responses of renal epithelial cells harboring an oncogenic KRAS mutant to characterize the phenotypic and molecular consequences of PAI-1 dysregulation. Engineered PAI-1 expression correlated with acquisition of a highly motile, growth-restricted mesenchymal phenotype with increased p-p53 levels and redistribution of the growth arrest p21 protein from a perinuclear to cytoplasmic localization. Collectively, these findings highlight PAI-1 as a potential driver of mesenchymal cell traits in KRAS-transformed renal epithelial cells.

## Data Availability

Detailed experimental protocols and information will be made available to interested researchers upon request.

## Supplementary Materials

The following supporting information can be downloaded at: https://www.mdpi.com/article/10.3390/biom16101517/s1, File S1: Available uncropped western blots.

## Abbreviations

SERPINE1, serine protease inhibitor, clade E, member 1; TME, tumor microenvironment; uPA, urokinase plasminogen activator; uPAR, uPA receptor; EGFR, epidermal growth factor receptor; ECM, extracellular matrix.
