# Supplementary material for: Engineered SERPINE1 Overexpression Activates the pp53/p21 Pathway and Promotes a Growth-Restricted, Migratory Phenotype in KRAS-Transformed Renal Proximal Tubular Cells
Source: Biomolecules. 2026 Oct 19;16(10):1517. doi: 10.3390/biom16101517 (PMC13702368; doi:10.3390/biom16101517)
Supplement: Supplementary file 1 [file biomolecules-16-01517-s001.zip › biomolecules-4551954-supplementary.pdf]

Figure 1B

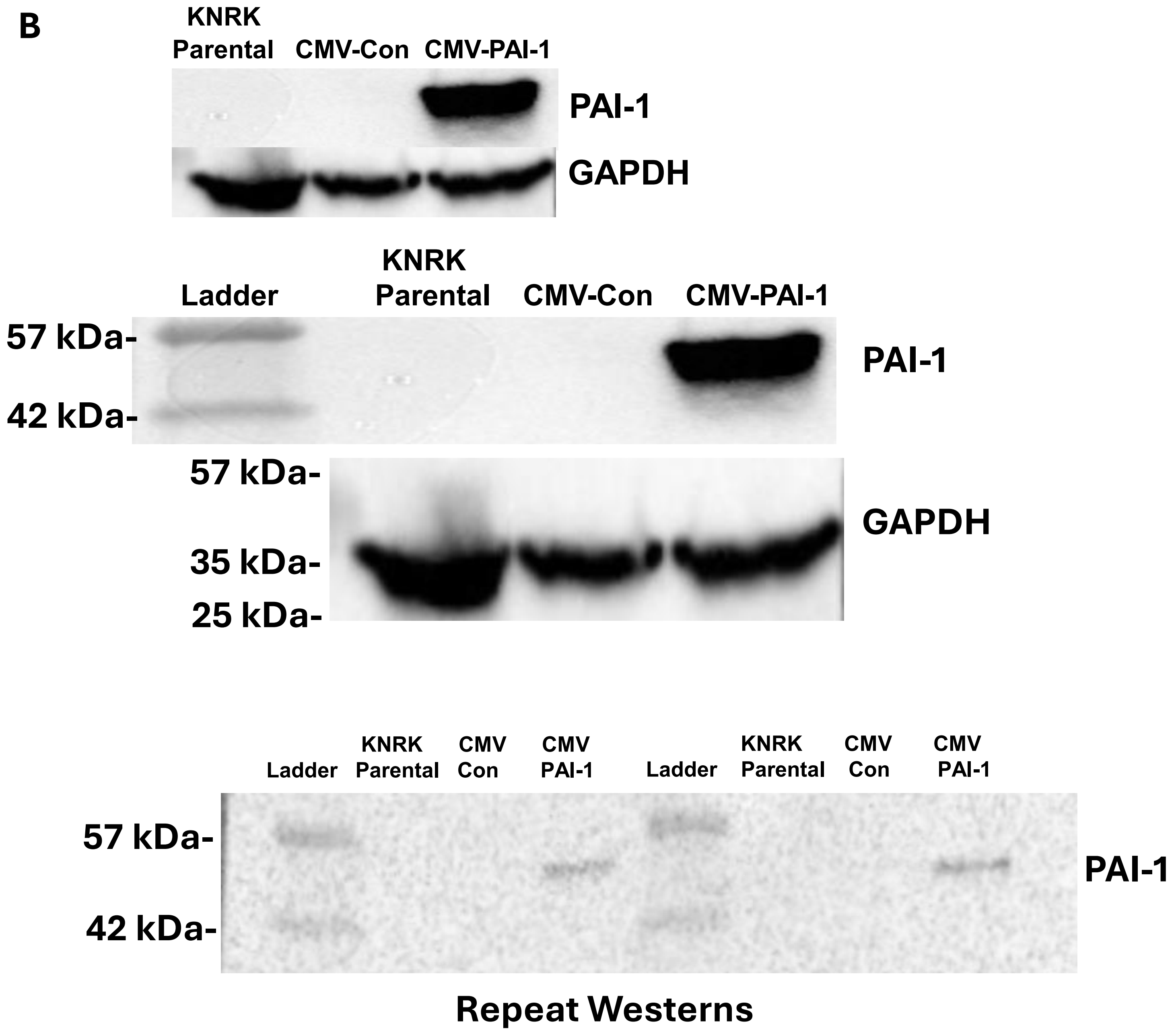
B
KNRK Parental
CMV-Con
CMV-PAI-1
PAI-1
GAPDH
Ladder
KNRK Parental
CMV-Con
CMV-PAI-1
57 kDa-
42 kDa-
PAI-1
57 kDa-
35 kDa-
25 kDa-
GAPDH
Ladder
KNRK Parental
CMV Con
CMV PAI-1
Ladder
KNRK Parental
CMV Con
CMV PAI-1
57 kDa-
42 kDa-
PAI-1
Repeat Westerns

Full Length Uncropped Blots

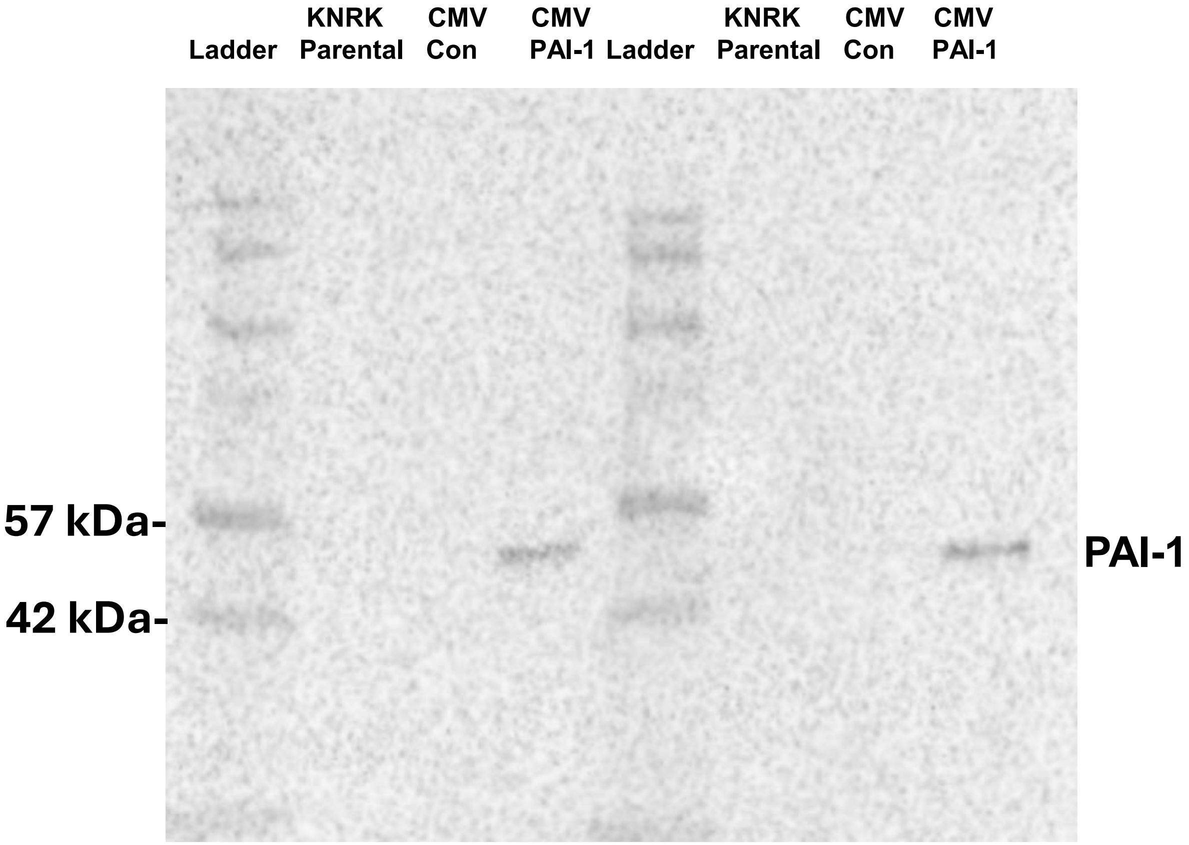

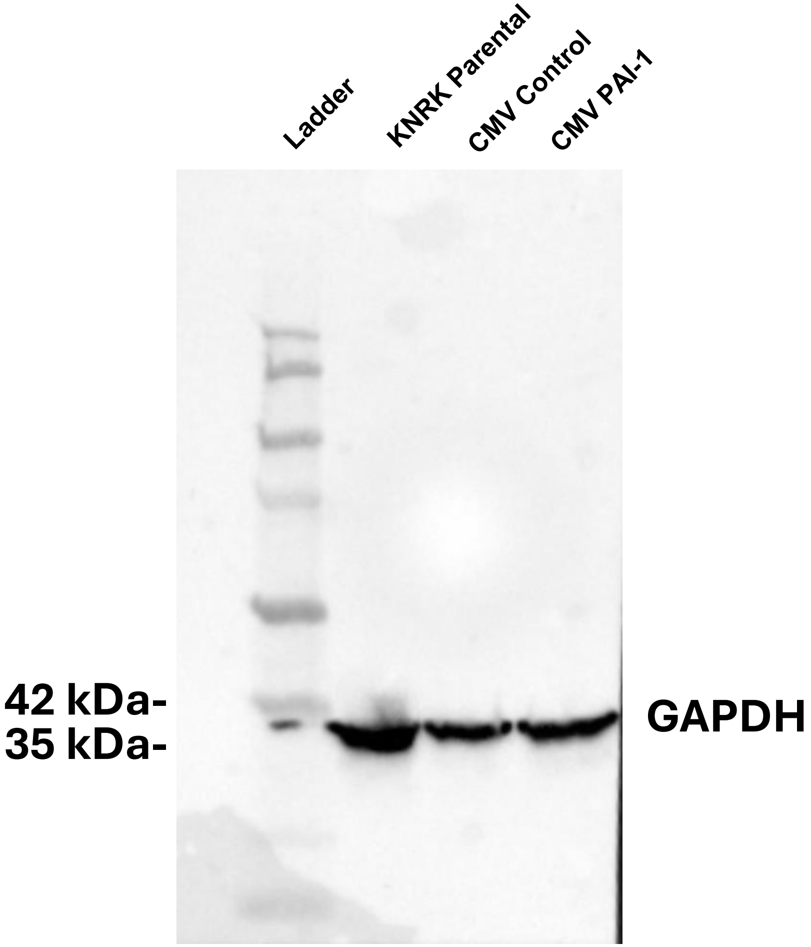

Figure 3A

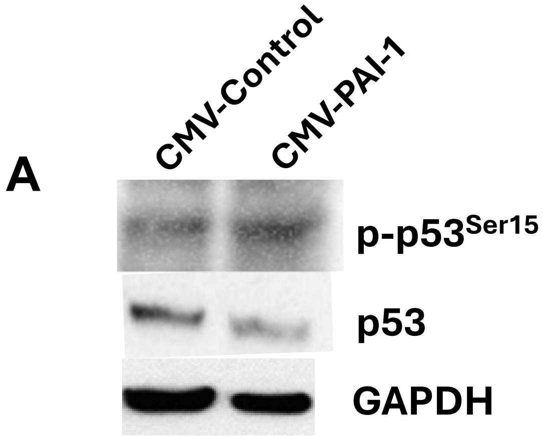

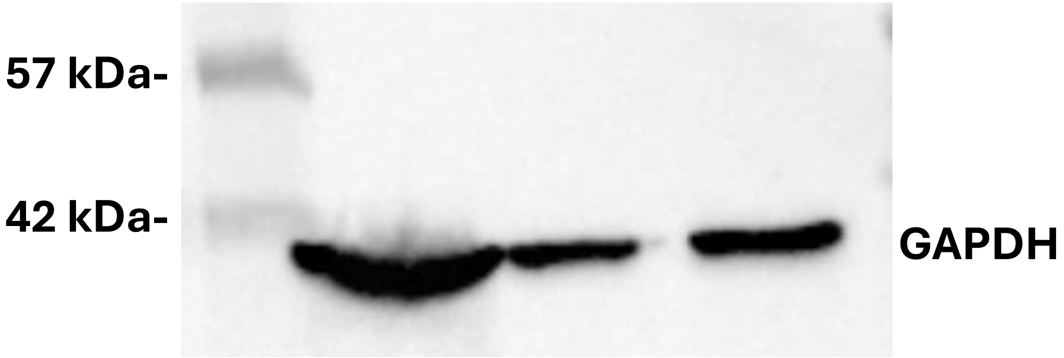

Uncropped additional GAPDH blot to illustrate specificity of the antibody

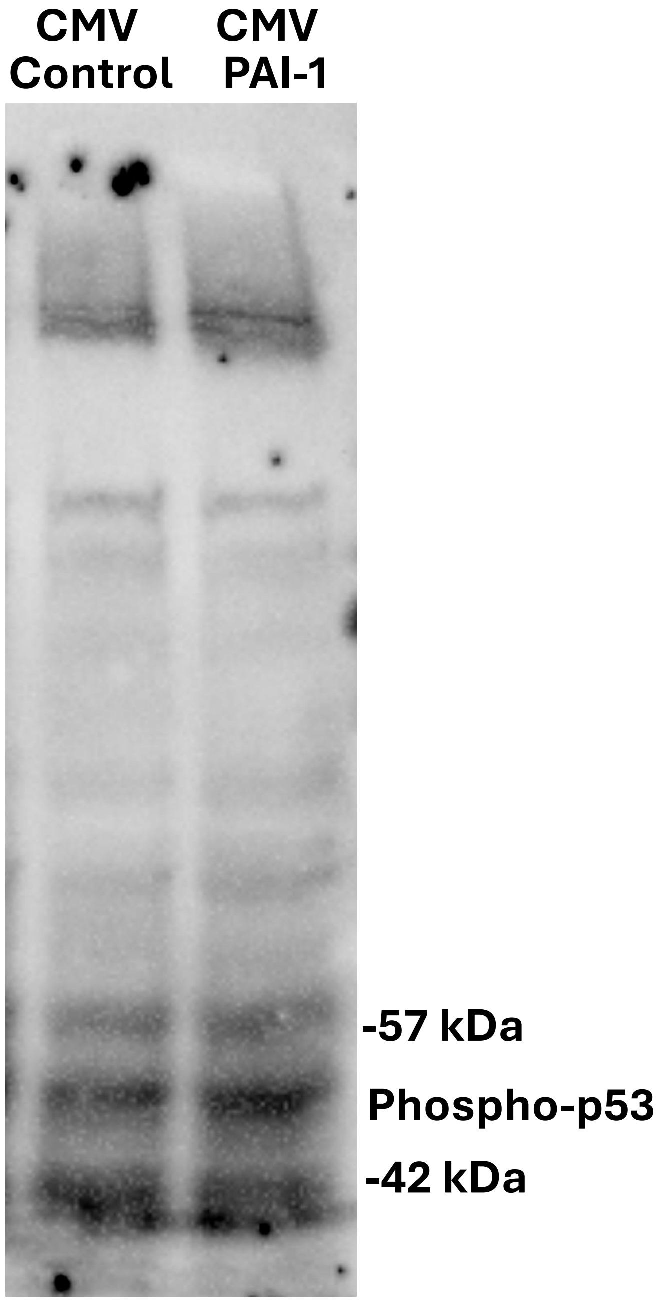

Uncropped p-p53ser15 blot in Fig. 3A
